# Retraction: Down-regulated LncR-MALAT1 suppressed cell proliferation and migration by inactivating autophagy in bladder cancer

**DOI:** 10.1039/d2ra90096c

**Published:** 2022-09-30

**Authors:** Jiude Qi, Yanfeng Chu, Guangyan Zhang, Hongjun Li, Dongdong Yang, Qi Wang

**Affiliations:** Department of Oncology, People’s Hospital of Laiwu Shandong 271100 China; Department of Laboratory, Yantaishan Hospital Shandong 264000 China; Department of Laboratory, People’s Hospital of Zhangqiu District Shandong 250200 China; Department of EGG Laboratory, Traditional Chinese Medicine Hospital of Zhangqiu Dirtrict Shandong 250200 China; Department of Nursing, People’s Hospital of Zhangqiu District Shandong 250000 China; Department of Urology, Qingdao Municipal Hospital No. 5 Donghai Middle Road, Shinan District Qingdao Shandong 266001 China wangqisdqd@163.com +86-0523-82789159

## Abstract

Retraction of ‘Down-regulated LncR-MALAT1 suppressed cell proliferation and migration by inactivating autophagy in bladder cancer’ by Jiude Qi *et al.*, *RSC Adv.*, 2018, **8**, 31019–31027, https://doi.org/10.1039/C8RA04876B.

The Royal Society of Chemistry hereby wholly retracts this *RSC Advances* article due to concerns with the reliability of the data.

In Fig. 2D, the ‘HT-1376/Control group’, ‘HT1376/MALAT1 scramble’, ‘253J/Control group’ and ‘253J/MALAT1 scramble’ panels all contain the same duplicated section, which shows that all 4 images come from the same image.

There are unexpected similarities within the background of the GAPDH panel in Fig. 2F, indicating that the bands may have been placed onto a false background.

The authors were asked to provide the raw data for this article, but did not respond. Given the significance of the concerns about the validity of the data, and the lack of raw data, the findings presented in this article are not reliable.

The authors were informed but have not responded to any correspondence regarding the retraction.

Signed: Laura Fisher, Executive Editor, *RSC Advances*

Date: 18th August 2022

## Supplementary Material

